# Development of a MicroRNA Signature Predictive of Recurrence and Survival in Pancreatic Ductal Adenocarcinoma

**DOI:** 10.3390/cancers13205168

**Published:** 2021-10-15

**Authors:** Nikhil T. Sebastian, Amy Webb, Kenneth W. Merrell, Eugene J. Koay, Adam R. Wolfe, Lizhi Zhang, Tyler J. Wilhite, Dalia Elganainy, Ryan Robb, Wei Chen, Jordan Cloyd, Mary Dillhoff, Allan Tsung, Laith Abushahin, Anne Noonan, Terence M. Williams

**Affiliations:** 1 Department of Radiation Oncology, Emory University Winship Cancer Institute, 1365 Clifton Rd, Atlanta, GA 30322, USA; nikhil.sebastian@emory.edu; 2 Department of Biomedical Informatics, The Ohio State University College of Medicine, 320 Lincoln Tower, 1800 Cannon Drive, Columbus, OH 43210, USA; Amy.Hite@osumc.edu; 3 Department of Radiation Oncology, Mayo Clinic, 200 First St SW, Rochester, MN 55090, USA; Merrell.Kenneth@mayo.edu; 4 Department of Radiation Oncology, The University of Texas MD Anderson Cancer Center, 1400 Pressler St, Houston, TX 77030, USA; EKoay@mdanderson.org (E.J.K.); dalia_elganainy@dfci.harvard.edu (D.E.); 5 Department of Radiation Oncology, The Winthrop P. Rockefeller Cancer Institute, The University of Arkansas for Medical Sciences, Little Rock, AR 72205, USA; 6 Division of Anatomic Pathology, Mayo Clinic, 200 First St SW, Rochester, MN 55905, USA; zhang.lizhi@mayo.edu; 7 Department of Radiation Oncology, UPMC Hillman Cancer Center-Shadyside, 55230 Centre Ave, Pittsburgh, PA 15232, USA; Wilhitetj2@upmc.edu; 8 Department of Pathology, University of North Carolina, Chapel Hill, NC 27599, USA; 9 Department of Pathology, The Ohio State University, 450 W. 10th Ave, Columbus, OH 43210, USA; wei.chen2@osumc.edu; 10 Division of Surgical Oncology, Department of Surgery, The Ohio State University Comprehensive Cancer Center—Arthur G. James Cancer Hospital and Richard J. Solove Research Institute, 460 W. 10th Ave, Columbus, OH 43210, USA; jordan.cloyd@osumc.edu (J.C.); mary.dillhoff@osumc.edu (M.D.); allan.tsung@osumc.edu (A.T.); 11 Division of Medical Oncology, Department of Internal Medicine, The Ohio State University Comprehensive Cancer Center—Arthur G. James Cancer Hospital and Richard J. Solove Research Institute, 460 W. 10th Ave, Columbus, OH 43210, USA; laith.abushahin@osumc.edu (L.A.); anne.noonan@osumc.edu (A.N.); 12 Department of Radiation Oncology, City of Hope National Medical Center, 1500 E. Duarte Rd, Duarte, CA 91010, USA

**Keywords:** pancreatic cancer, microRNA, locoregional recurrence, local recurrence, adjuvant radiation, neoadjuvant radiation

## Abstract

**Simple Summary:**

Optimal patient selection for radiotherapy in pancreatic cancer is unestablished and may be improved with molecular profiling. To this end, we developed and validated a microRNA signature that predicted for worse locoregional recurrence and overall survival in patients with resectable pancreatic cancer. In a separate cohort of patients with borderline resectable and locally advanced pancreatic cancer, this risk signature was also predictive of worse locoregional recurrence, distant recurrence, and overall survival. Additionally, borderline resectable or locally advanced patients who had high risk score and did not receive radiation had worse outcomes compared to patients who either had low risk score or received radiation, irrespective of risk score. This risk signature may be useful in assessing patient prognosis and tailor therapy in patients with resectable, borderline resectable, or locally advanced pancreatic cancer, but requires further study.

**Abstract:**

Background: Optimal patient selection for radiotherapy in pancreatic ductal adenocarcinoma (PDAC) is unestablished. Molecular profiling may select patients at high risk for locoregional recurrence (LRR) who would benefit from radiation. Methods: We included resectable pancreatic cancer (R-PDAC) patients, divided into training and validation cohorts, treated among three institutions with surgery and adjuvant chemotherapy, and borderline resectable or locally advanced pancreatic cancer (BR/LA-PDAC) patients treated with chemotherapy with or without radiation at the primary study institution. We isolated RNA from R-PDAC surgical specimens. Using NanoString, we identified miRNAs differentially expressed between normal and malignant pancreatic tissue. ElasticNet regression identified two miRNAs most predictive of LRR in the training cohort, miR-181b/d and miR-575, which were used to generate a risk score (RS). We evaluated the association of the median-dichotomized RS with recurrence and overall survival (OS). Results: We identified 183 R-PDAC and 77 BR/LA-PDAC patients with median follow up of 37 months treated between 2001 and 2014. On multivariable analysis of the R-PDAC training cohort (*n* = 90), RS was associated with worse LRR (HR = 1.34; 95%CI 1.27–11.38; *p* = 0.017) and OS (HR = 2.89; 95%CI 1.10–4.76; *p* = 0.027). In the R-PDAC validation cohort, RS was associated with worse LRR (HR = 2.39; 95%CI 1.03–5.54; *p* = 0.042), but not OS (*p* = 0.087). For BR/LA-PDAC, RS was associated with worse LRR (HR = 2.71; 95%CI 1.14–6.48; *p* = 0.025), DR (HR = 1.93; 95%CI 1.10–3.38; *p* = 0.022), and OS (HR = 1.97; 95%CI 1.17–3.34; *p* = 0.011). Additionally, after stratifying by RS and receipt of radiation in BR/LA-PDAC patients, high RS patients who did not receive radiation had worse LRR (*p* = 0.018), DR (*p =* 0.006), and OS (*p* < 0.001) compared to patients with either low RS or patients who received radiation, irrespective of RS. Conclusions: RS predicted worse LRR and OS in R-PDAC and worse LRR, DR, and OS in BR/LA-PDAC. This may select patients who would benefit from radiation and should be validated prospectively.

## 1. Introduction

Pancreatic ductal adenocarcinoma (PDAC) is the third leading cause of cancer mortality in the United States, accounting for over 45,000 deaths annually [1]. PDAC is typified by high rates of local (e.g., primary tumor or tumor bed), regional (e.g., lymph nodes), and distant recurrence after surgery. While surgical resection offers the best chance for long-term outcomes, many patients do not initially present with resectable disease, but rather with borderline-resectable, locally advanced (unresectable), or metastatic disease. For resectable disease, the role of postoperative radiotherapy is controversial, given the uncertain benefit in overall survival in randomized trials [2,3,4]. The role of radiotherapy is similarly controversial in patients with borderline or locally advanced PDAC [5,6]. Despite the equivocal benefit of radiotherapy on survival in cohorts of unselected patients, it is clear that radiotherapy improves locoregional control [2,3,4,5]. However, predicting which patients might be at increased risk of locoregional failure is critical to determining whether certain subsets of patients benefit from additional locoregional therapy, given the potential toxicity from radiation. Molecular personalized assessment may allow for improved selection of patients who would benefit from radiotherapy [7].

Molecular assays predictive of recurrence are utilized in treatment selection for other malignancies with great clinical success [8,9]. Identification of novel biomarkers may aid in the prognostication or tailoring of treatment or follow up. Specifically, microRNAs (miRNAs) have emerged as valuable biomarkers; these non-coding RNAs are instrumental in post-transcriptional regulation of gene expression and interact with target mRNAs to induce their degradation. Their dysregulation has been identified in numerous cancers, and they are of particular interest in PDAC given their association with tumor invasiveness and treatment resistance [10]. Multiple miRNA expression profiling studies have established differential expression of miRNAs between normal and malignant pancreatic tissue [11]. These miRNAs have been implicated in pancreatic cancer progression through a variety of mechanisms, including negative regulation of tumor suppressor genes (e.g., miR-21-mediated downregulation of PTEN), underexpression of tumor suppressing miRNAs (e.g., miR-124 negative regulation of Rac1 oncogene), or cell cycle alteration resulting in enhanced proliferation (e.g., miR-203 downregulation inducing G1 phase progression) [12]. In a previous single institution pilot study, we assessed the prognostic capacity of miRNAs and developed a tentative signature derived from a small cohort of patients [13]. In this current study, we sought to identify and then validate a revised miRNA signature predictive of locoregional recurrence in a cohort of patients with resected PDAC derived from a large, multi-institutional cohort. In a secondary analysis, we sought to confirm the predictive and prognostic utility of the miRNA signature in a cohort of patients with borderline resectable or locally advanced PDAC treated at our institution.

## 2. Methods

### 2.1. Patient Selection and Endpoints

This study was approved by the institutional review boards of all participating institutions (IRB protocol 2014C0077 (OSU-14093), opened in 2014). For patients with resected PDAC, we obtained core samples of formalin-fixed, paraffin-embedded (FFPE) tumor and adjacent normal tissue from patients who underwent surgery and adjuvant chemotherapy at Ohio State University, Mayo Clinic, or MD Anderson. For patients with borderline resectable or locally advanced PDAC, FFPE tumors from pretreatment core biopsy samples were obtained. All patients with borderline resectable or locally advanced PDAC were treated with definitive intent at Ohio State University with chemotherapy, with or without radiotherapy or surgery.

Recurrence was defined radiologically using computed tomography (CT) scans of the abdomen and pelvis during follow up, which was performed every 3–6 months after completion of treatment. Locoregional (local and regional) recurrence was defined as either pathologically confirmed recurrence within the surgical bed (for resected PDAC), primary tumor (for borderline-resectable or locally advanced PDAC), or lymph nodes occurring in standard post-operative radiation treatment fields, or as measurable progression of disease in these regions on 2 consecutive scans per RECIST version 1.1 criteria [14] with corroborating rise in CA19-9. Distant recurrence was defined as recurrence outside of the surgical bed/pancreas, and regional lymph nodes. Overall survival was defined as the interval between the date of surgery (for resected PDAC) or first induction chemotherapy cycle (for borderline resectable and locally advanced PDAC) and the date of death or last follow up.

### 2.2. MiRNA Expression Profiling

For all samples, we used the Norgen FFPE RNA Isolation kit (Norgen Biotek, Thorold, ON, Canada) to collect total genomic RNA from viable, non-necrotic regions of tumor in FFPE blocks as defined by board-certified pathologists specializing in gastrointestinal pathology as previously described [15]. Expression profiling of miRNAs was performed using the nCounter Human v3 miRNA Expression Assay (NanoString Technologies, Seattle WA, USA). Counts were normalized with voom and limma R packages to perform differential expression analysis between groups of samples.

A miRNA was removed if more than 90% of the samples had log counts less than the negative background. Negative background was calculated as the mean of the log2 negative background counts plus 1.5 times the standard deviation. A sample was removed if more than 70% of miRNA probes fell below the background cutoff for the Ohio State University cohort and 60% for the Mayo Clinic and MD Anderson cohorts. Final sample counts were 69, 26, and 88 for Mayo Clinic, MD Anderson, and Ohio State University, respectively. The filtered data were normalized by the geometric mean and log2 transformed.

With mixed modeling we identified miRNAs differentially expressed in tumor and normal tissue of resected PDAC patients. Using the R CrossValidate package, we created equal-sized training and validation cohorts of patients with resected PDAC balanced by institution and locoregional recurrence events. In the training cohort there were 43, 34, and 13 patients, and in the validation cohort there were 45, 35, and 13 patients treated at Ohio State University, Mayo Clinic, and MD Anderson Cancer Center, respectively. Using the pool of miRNAs distinguishing tumor from normal, ElasticNet regression was used to select predictive groups of miRNAs and identified two miRNAs (miR-181b/d and miR-575) predictive of locoregional recurrence (handled as a censored variable) and overall survival in the training dataset. Beta coefficients, generated by ElasticNet, were used to calculate a risk score by summing the product of each miRNA expression level and its beta coefficient, as follows:

−1.8142*hsa.miR.181b.5p/hsa.miR.181d.5p − 0.1856*hsa.miR.575

We studied the miRNA risk score both as a continuous variable and as a variable dichotomized by the respective median of each cohort and evaluated its association with locoregional recurrence, distant recurrence, and overall survival using Cox proportional hazards and Kaplan–Meier analysis. For resectable PDAC patients, we accounted for age, pathologic T and N stage, histologic grade, postoperative CA 19-9, and surgical margin status in the Cox model. For borderline resectable or locally advanced patients, we accounted for age, clinical T and N stage, receipt of radiation, and surgical resection status. All statistical analyses were performed using R version 3.5.0 and 3.4.3 (R Project for Statistical Computing).

## 3. Results

### 3.1. Resectable Pancreatic Cancer

We identified a total of 183 resectable PDAC patients who underwent surgical resection followed by chemotherapy. The median follow up of all patients was 20.9 months (interquartile range (IQR) 13.6–33.3), and the median follow up of living patients was 37.1 months (IQR 25.3–55.3). Of these, 90 patients were assigned to the training cohort and 93 were assigned to the validation cohort (Table 1).

**Figure 1:**
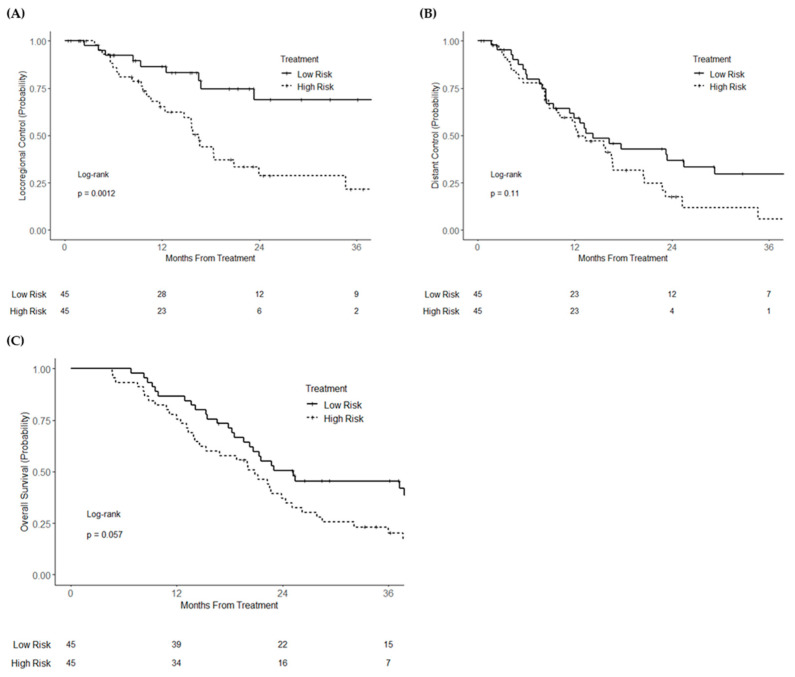
Kaplan–Meier curves of (**A**) locoregional recurrence, (**B**) distant recurrence, and (**C**) overall survival of the training cohort of resectable pancreatic cancer patients, stratified by risk score group.

In the training cohort (*n* = 90), when analyzed as a continuous variable, the miR risk score was associated with locoregional recurrence (HR = 1.43; 95% CI 1.14–1.81; *p* = 0.002), but not distant recurrence (HR = 1.11; 95% CI 0.92–1.34; *p* = 0.28) or overall survival (HR = 1.07; 95% CI 0.91–1.25; *p* = 0.44). After dichotomization, high miR risk score was associated with increased locoregional recurrence (HR = 3.29; 95% CI 1.53–7.04; *p* = 0.002), but not distant recurrence (HR = 1.52; 95% CI 0.90–2.54; *p* = 0.11). Overall survival was numerically worse but statistically non-significant in the high-risk group (HR = 1.58; 95% CI 0.98–2.54; *p* = 0.059). Three-year locoregional control was 68.8% and 21.5% (logrank *p* = 0.0012) for high- and low-risk groups, respectively, and 3-year overall survival was 45.5% and 20.3% (logrank *p* = 0.057), respectively (Figure 1A–C). On multivariable analysis, high risk score continued to be independently associated with locoregional recurrence (HR = 1.34; 95% CI 1.27–11.38; *p* = 0.017) (Table S1). A higher miR risk score was also significantly associated with worse overall survival (HR = 2.89; 95% CI 1.10–4.76; *p* = 0.027), despite no significant association with distant recurrence (HR = 1.62; 95% CI 0.75–1.50; *p* = 0.22). In an analysis including receipt of radiation as a covariable (five patients received radiation), binary risk score remained associated with locoregional recurrence (HR = 5.66; 95% CI 1.62–19.77; *p* = 0.007) and overall survival (HR = 2.40; 95% CI 1.12–5.16; *p* = 0.025), and was not associated with distant recurrence (HR = 1.86; 95% CI 0.82–4.22; *p* = 0.14). Finally, in a secondary multivariable analysis excluding the five patients who received adjuvant radiation in the training cohort, binary risk score remained associated with locoregional recurrence (HR = 4.57; 95% CI 1.35–15.49; *p* = 0.015) and overall survival (HR = 2.29; 1.08–4.89; *p* = 0.031).

**Figure 2:**
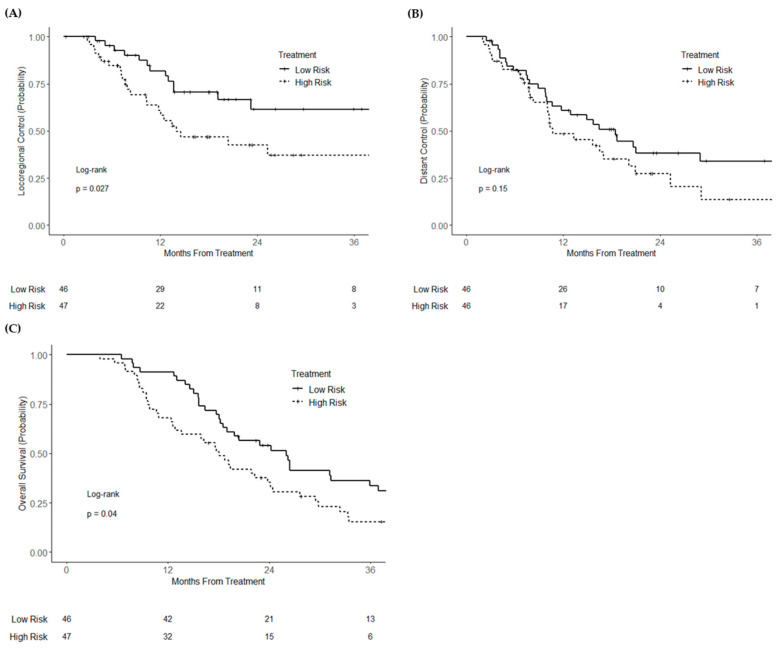
Kaplan–Meier curves of (**A**) locoregional control, (**B**) distant control, and (**C**) overall survival of the validation cohort of resectable pancreatic cancer patients, stratified by risk score group.

In the validation cohort (*n* = 93), high miR risk score (analyzed as a continuous variable) was associated with worse locoregional recurrence (HR = 1.27; 95% CI 1.00–1.60; *p* = 0.046) and overall mortality (HR = 1.21; 95% CI 1.01–1.44; *p* = 0.037) on univariable analysis. There was no significant association with distant recurrence (HR = 1.05; 95% CI 0.86–1.29; *p* = 0.65). After dichotomization, high miR risk score was associated with worse locoregional recurrence (HR = 2.08; 95% CI 1.07–4.03; *p* = 0.030) and overall survival (HR = 1.61; 95% CI 1.02–2.54; *p* = 0.042), but not distant recurrence (HR = 1.48; 95% CI 0.87–2.52; *p* = 0.15). Three-year locoregional control was 61.5% and 37.2% (logrank *p* = 0.027) for high- and low-risk groups, respectively, and 3-year overall survival was 33.5% and 15.0% (logrank *p* = 0.040), respectively (Figure 2A–C). On multivariable analysis, high miR risk score remained associated with increased locoregional recurrence (HR = 2.39; 95% CI 1.03–5.54; *p* = 0.042) (Table S2), but not distant recurrence (HR = 1.83; 95% CI 0.78–4.30; *p* = 0.16). High risk score was numerically associated with worse overall survival, although this was statistically non-significant (HR = 1.76; 95% CI 0.92–3.36; *p* = 0.087). In a secondary multivariable analysis excluding the two patients who received adjuvant radiation in the validation cohort, binary risk score trended toward significance for association with locoregional recurrence (HR = 2.04; 95% CI 0.88–4.75; *p* = 0.097) but was significantly associated with worse overall survival (HR = 1.59; 95% CI 0.84–3.04; *p* = 0.016).

### 3.2. Borderline Resectable/Unresectable Pancreatic Cancer

To evaluate the applicability of the miR risk signature to the neoadjuvant (borderline resectable) or inoperable setting (locally advanced), we identified a total of 77 patients with borderline resectable or locally advanced PDAC treated with chemotherapy with or without radiotherapy (Table S3). The median follow up of all patients was 13.8 months (IQR 11.1–31.5), and the median follow up of living patients was 37.3 months (33.5–42.7).

**Figure 3:**
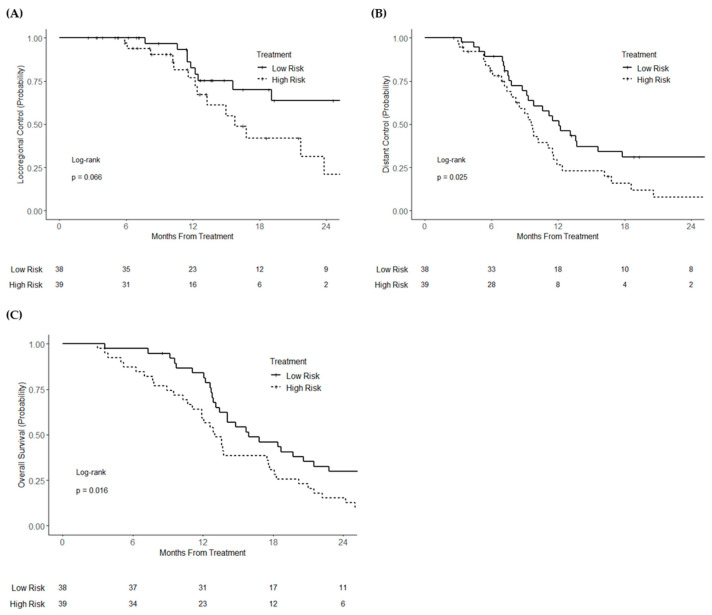
Kaplan–Meier curves of (**A**) locoregional control, (**B**) distant control, and (**C**) overall survival of patients with borderline resectable or locally advanced (unresectable) pancreatic cancer, stratified by risk score group.

On univariable analysis, continuous miR risk score was associated with worse overall survival (HR = 1.32; 95% CI 1.04–1.67; *p* = 0.025) and distant recurrence (HR = 1.32; 95% CI 1.04–1.69; *p* = 0.025). High miR risk score was associated with statistically non-significant increased locoregional recurrence (HR = 1.44; 95% CI 0.97–2.14; *p* = 0.072). After dichotomization, high binary miR risk score was associated with worse overall survival (HR = 1.79; 95% CI 1.11–2.90; *p* = 0.018) and worse distant recurrence (HR = 1.84; 95% CI 1.07–3.15; *p* = 0.027), and had a statistically non-significant association with higher locoregional recurrence (HR = 2.09; 95% CI 0.94–4.66; *p* = 0.073) (Figure 3A–C). On multivariable analysis, high risk score was associated with worse locoregional recurrence (HR = 2.71; 95% CI 1.14–6.48; *p* = 0.025) (Table S4), distant recurrence (HR = 1.93; 95% CI 1.10–3.38; *p* = 0.022), and overall survival (HR = 1.97; 95% CI 1.17–3.34; *p* = 0.011).

**Figure 4:**
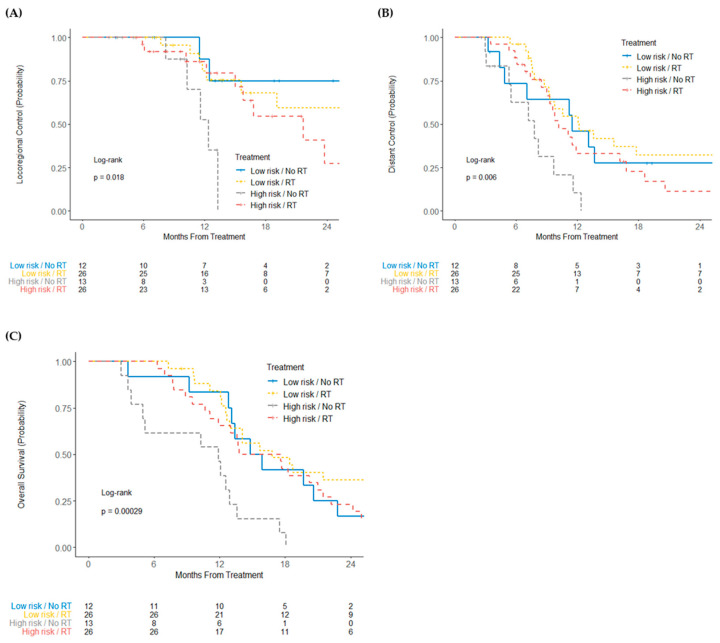
Kaplan–Meier curves of (**A**) locoregional control, (**B**) distant control, and (**C**) overall survival of patients with unresectable or borderline resectable pancreatic cancer, stratified by risk score group and receipt of radiation.

After stratification for risk score and receipt of radiotherapy, high-risk patients who did not receive radiotherapy had worse locoregional recurrence (log-rank *p* = 0.018), distant recurrence (log-rank *p* = 0.006), and overall survival (log-rank *p* = 0.00029) (Figure 4).

## 4. Discussion

We developed and validated a multi-miRNA risk score associated with higher risk of locoregional recurrence in a multi-institutional cohort of patients with resected PDAC. In addition, high miR risk score was frequently associated not only with locoregional recurrence, but also decreased survival. Furthermore, we found this miR risk score was associated with higher rates of locororegional recurrence, distant recurrence, and worse overall mortality in our single-institution cohort of patients with borderline resectable or locally advanced disease treated with chemotherapy with or without radiation. This is, to our knowledge, the first validated, molecular-based risk score developed for PDAC that can predict locoregional recurrence and survival.

During the past two decades, significant work has been undertaken to identify diverse molecular biomarkers for PDAC, but these studies have been hampered by small sample sizes and biomarker non-specificity [16]. MicroRNAs are small non-coding RNAs involved in post-transcriptional regulation of gene expression, and they have emerged as pleiotropic, highly dysregulated biomarkers in PDAC. We previously demonstrated the feasibility of using microRNAs as prognostic markers in resected PDAC using microRNAs with a demonstrated link to PDAC in the literature. That study was limited due to the single institution design, smaller scope of analyzed microRNAs, and lack of patterns of failure data in validation datasets [13]. In our current study, we evaluated microRNAs most prognostic in a multi-institutional dataset containing survival and patterns of failure data out of all available analyzable microRNAs, thus allowing for a potentially more accurate and generalizable panel. This resulted in the distinct microRNA panel identified in our current study, which identifies a correlation between lower expression of miR-181b/d and miR-575 with increased risk of recurrence. Of these, miR-181b/d has been established as being dysregulated in PDAC versus normal pancreatic tissue and has been linked to gemcitabine resistance [17,18]. The exact functions of the miR-181 family in PDAC are unclear, and its role in other malignancies is heterogeneous, being implicated in oncogenesis, cell proliferation, and cell migration by affecting a number of targets [19,20], which could theoretically promote malignant transformation of PDAC and treatment resistance. Additionally, miR-181b has implicated in enhancing chemosensitivity in non-small cell lung cancer through inactivation of the TGFβR1/Smad signaling pathway [21]. It is possible that decreased expression of miR-181b promotes hyperactivation of this pathway and confers gemcitabine resistance [22]. Additionally, miR-181d has been associated with tumor suppressive effects in non-small cell lung cancer, as well as in gastric cancer through a variety of targets including PI3K/AKT, a key mediator of disease progression [23,24]. Our findings are consistent with miR-181b/d potentially having tumor suppressive roles in PDAC. Although miR-575 has not been previously linked to PDAC, it has been implicated in development of other gastrointestinal cancers, such as gastric cancer via inhibition of PTEN [25], biliary cancer via inhibition of p27Kip1 [26], and hepatocellular carcinoma via inhibition of ST7L [27]. Interestingly, in our study, miR-575 decreased expression was associated with higher risk score and recurrence; it is possible that miR-575 may have disparate tissue-specific functions and that its downregulation is secondary to upregulation of other pathways that mediate PDAC recurrence. Certainly, more studies are needed.

Although radiation was initially supported as adjuvant therapy after prospective evidence of a disease-free and overall survival benefit in a small randomized trial [2], the absence of benefit found in subsequent studies has called into question the benefit of routine adjuvant chemoradiotherapy for resected PDAC [3,4]. Rather, modern practice guidelines recommend consideration of postoperative radiotherapy only in the setting of positive margin (R1) resection [28]. While the role of postoperative chemoradiation is currently being evaluated in the recently completed Radiation Therapy Oncology Group 0848 phase III clinical trial, it is generally accepted that specific subgroups of patients with sufficiently high risk of locoregional recurrence may benefit the most from the addition of radiotherapy [29]. Indeed, many studies suggest potential survival benefit in administering radiation for patients with high-risk clinicopathologic features, such as pathologic lymph node involvement, positive surgical margin status, and elevated CA19-9 [30,31,32,33,34]. Despite increasing awareness of prognostic biomarkers in PDAC [16] and recent genomics-based studies to predict chemotherapeutic response [35,36], there is a dearth of molecular data regarding the utilization of radioresistance biomarkers to predict clinical response to radiotherapy. Given the equivocal role of postoperative radiotherapy for all patients with resected PDAC, which remains associated with high mortality despite improvements in adjuvant chemotherapy regimens [37], a molecular assay-based predictive panel may be useful in selecting subsets of patients for adjuvant radiation. Indeed, similar efforts have led to significant changes in the treatment paradigm for breast cancer, for which the use of molecular assays has become standard of care in the selection of patients for chemotherapy and may be valuable for selection of patients for radiation [38,39].

The role of radiotherapy is similarly controversial in locally advanced PDAC, for which the addition of radiation to chemotherapy has not shown an overall survival benefit, with few exceptions [40,41], in the majority of randomized trials [5,42,43,44]. Additionally, a clear survival benefit to radiotherapy has not been established in patients with resectable or borderline resectable PDAC, as evident in the recent PREOPANC phase III trial (which randomized patients to neoadjuvant chemoradiation versus upfront surgery), despite improvements in lymph node downstaging, reduction in margin positivity rate, and improved disease control from chemoradiotherapy [45]. In the present study, we observed the risk score derived from patients with resected PDAC was also found to be predictive of recurrence and mortality in a cohort of patients who did not receive up front surgery (i.e., borderline-resectable and locally advanced PDAC). Importantly, we found similar locoregional control in patients with a high-risk miRNA score who received radiation and patients with a low-risk score (regardless of radiation), while patients with a high-risk score who did not receive radiotherapy had significantly worse locoregional control, distant recurrence rate, and overall survival (Figure 4). Distant control and overall survival were similarly improved in high-risk patients who received radiotherapy when compared to those who did not, with similar outcomes to those patients in the low-risk cohort. Taken together, selective radiotherapy for patients with high-risk resectable, borderline resectable, or locally advanced PDAC, as determined by the miR risk score, may improve outcomes. Conversely, withholding radiotherapy for low-risk miR risk score patients may spare them unnecessary side effects of radiotherapy.

Strengths of this study include validation on an independent dataset as well as this study representing the largest miRNA profiling effort performed in pancreatic cancer (*n* = 183 patients), with high-quality, clinically annotated data. In addition, the data were acquired from three independent institutions, representing heterogeneous groups of patients that likely better represent the larger patient population diagnosed with PDAC in the U.S and worldwide (i.e., rather than focusing on a single institution). Furthermore, the use of NanoString miRNA profiling platform has advantages that will facilitate broader clinical translation, in that low-input RNA is required, does not require amplification steps by virtue of utilizing direct hybridization, does not require technical replicates, and exhibits high concordance between FFPE and frozen tumor samples. Limitations of the study should also be noted. The study was retrospective in nature and did not include data from prospectively collected patient samples nor from large clinical trials. In addition, the data from borderline resectable and locally advanced PDAC in this study are derived from a single institution with limited patient numbers, and they should be interpreted more cautiously until there is independent validation of the miRNA risk signature in predicting recurrence and mortality in this group of patients.

In conclusion, we developed and validated a promising multi-miRNA risk signature predictive of locoregional recurrence and overall mortality in a multi-institutional cohort of patients with resectable PDAC, and we also validated it an institutional cohort of patients with borderline resectable or locally advanced PDAC. In a secondary analysis of the patients with borderline resectable or locally advanced PDAC (of which about two-thirds received radiation), we found that patients with a high-risk miR score who received radiotherapy likewise had similar locoregional control, distant control, and overall survival as patients with a low-risk score, whereas patients with a high-risk score who did not receive radiotherapy had significantly worse outcomes. This risk score should be further validated prospectively and may be useful in prognosticating PDAC patients and selecting those patients who would benefit from treatment intensification with addition of radiotherapy either adjuvantly or neoadjuvantly.

## Figures and Tables

**Table 1:** Patient and disease characteristics of patients with resectable pancreatic cancer in the training and validation cohorts.

Variable	Training Cohort			Validation Cohort		
	Low Risk (*n* = 45)	High Risk (*n* = 45)	*p*	Low Risk (*n* = 46)	High Risk (*n* = 47)	*p*
Age (years)						
<60 ≥60	15 (33.3%) 30 (66.7%)	12 (26.7%) 33 (73.3%)	0.65	15 (32.6%) 31 (67.4%)	13 (27.7%) 34 (72.3%)	0.60
Sex						
Male Female	24 (53.3%) 21 (46.7%)	27 (60.0%) 18 (40.0%)	0.67	25 (54.3%) 21 (45.7%)	28 (59.6%) 19 (40.4%)	0.61
Pathologic T stage						
1–2 3–4	12 (26.7%) 33 (73.3%)	4 (8.9%) 41 (91.1%)	0.05	8 (17.4%) 38 (82.6%)	4 (8.5%) 43 (91.5%)	0.20
Pathologic N stage						
0 1	14 (31.1%) 31 (68.9%)	9 (20.0%) 36 (80.0%)	0.33	17 (37.0%) 29 (63.0%)	8 (17.0%) 39 (83.0%)	0.03
Margins						
Negative Positive	35 (77.8%) 10 (22.2%)	17 (37.8%) 28 (62.2%)	<0.001	37 (80.4%) 9 (19.6%)	16 (34.0%) 31 (66.0%)	<0.001
Grade						
1–2 3	19 (42.2%) 26 (57.8%)	28 (62.2%) 17 (37.8%)	0.09	18 (39.1%) 28 (60.9%)	27 (57.4%) 20 (42.6%)	0.08
Post-op CA 19–9						
≤90 >90 Unavailable	30 (66.7%) 7 (15.6%) 8 (17.8%)	24 (21.0%) 16 (35.6%) 16 (35.6%)	1.00	27 (58.7%) 3 (6.5%) 16 (34.8%)	27 (57.4%) 9 (19.1%) 11 (23.4%)	0.12
Locoregional recurrence						
No Yes	36 (80.0%) 9 (20.0%)	18 (40.0%) 27 (60.0%)	<0.001	32 (69.6%) 14 (30.4%)	23 (48.9%) 24 (51.1%)	0.04

## Data Availability

Data can be made available upon request to the corresponding author.

## Supplementary Materials

The following are available online at https://www.mdpi.com/article/10.3390/cancers13205168/s1, Table S1: Multivariable analysis for locoregional recurrence in the training cohort of resectable pancreatic cancer patients, Table S2: Multivariable analysis for locoregional recurrence of the validation cohort of resectable pancreatic cancer patients, Table S3: Patient and disease characteristics of patients with borderline resectable or locally advanced pancreatic cancer, Table S4: Multivariable analysis for locoregional recurrence of patients with borderline resectable or locally advanced pancreatic cancer.
