# Supplementary material for: Development of a MicroRNA Signature Predictive of Recurrence and Survival in Pancreatic Ductal Adenocarcinoma
Source: Cancers (Basel). 2021 Oct 15;13(20):5168. doi: 10.3390/cancers13205168 (PMC8534163; doi:10.3390/cancers13205168)
Supplement: Supplementary file 1 [file cancers-13-05168-s001.zip › cancers-1366878-supplementary.pdf]

cancers

MDPI

Supplementary Materials: Development of a MicroRNA Signature Predictive of Recurrence and Survival in Pancreatic Ductal Adenocarcinoma

Nikhil T. Sebastian, Amy Webb, Kenneth W. Merrell, Eugene J. Koay, Adam R. Wolfe, Lizhi Zhang, Tyler J. Wilhite, Dalia Elganainy, Ryan Robb, Wei Chen, Jordan Cloyd, Mary Dillhoff, Allan Tsung, Laith Abushahin, Anne Noonan and Terence M. Williams

Table S1. Multivariable analysis for locoregional recurrence in the training cohort of resectable pancreatic cancer patients.

Variable	Hazard Ratio	*p*
Binary Risk Score		
Low Risk	1.0	--
High Risk	3.80 (1.27–11.38)	0.017
Age		
<60	1.0	--
≥60	0.59 (0.23–1.53)	0.28
Pathologic T stage		
1–2	1.0	--
3–4	3.42 (0.74–15.79)	0.12
Pathologic N stage		
0	1.0	--
1	1.39 (0.43–4.47)	0.58
Surgical margin		
Negative	1.0	--
Positive	1.45 (0.52–4.06)	0.48
Grade		
1–2	1.0	--
3	1.54 (0.67–3.58)	0.31
Post-op CA 19–9		
≤90	--	--
>90	2.27 (0.82–6.32)	0.11

Table S2. Multivariable analysis for locoregional recurrence of the validation cohort of resectable pancreatic cancer patients.

Variable	Hazard Ratio	*p*
Binary Risk Score		
Low Risk	1.0	--
High Risk	2.39 (1.03–5.54)	0.042
Age		
<60	1.0	--
≥60	0.46 (0.22–1.00)	0.049
Pathologic T stage		
1–2	1.0	--
3–4	2.20 (0.48–9.97)	0.31
Pathologic N stage		
0	1.0	--
1	2.35 (0.88–6.32)	0.31
Surgical margin		
Negative	1.0	--
Positive	1.32 (0.54–3.20)	0.54

Grade		
1–2	1.0	--
3	2.34 (1.03–5.30)	0.042
Post-op CA 19–9		
≤90	1.0	--
>90	2.35 (0.88–6.32)	0.089

Table S3. Patient and disease characteristics of patients with borderline resectable or locally advanced pancreatic cancer.

**Variable**	**Low Risk (*n* = 38)**	**High Risk (*n* = 39)**	***p***
Age			
<60	13 (34.2%)	12 (30.8%)	0.94
≥60	25 (65.8%)	27 (69.2%)	
Sex			
Male	16 (42.1%)	18 (46.2%)	0.90
Female	22 (57.9%)	21 (53.8%)	
Clinical T-stage			
1–2	12 (31.6%)	12 (30.8%)	1.00
3–4	26 (68.4%)	27 (69.2%)	
Clinical N stage			
0	28 (73.7%)	22 (56.4%)	0.18
1	10 (26.3%)	17 (43.6%)	
Radiation			
No	12 (31.6%)	13 (33.3%)	1.00
Yes	26 (68.4%)	26 (66.7%)	
Surgery			
No	11 (28.9%)	10 (25.6%)	0.94
Yes	27 (71.1%)	29 (74.4%)	

Table S4. Multivariable analysis for locoregional recurrence of patients with borderline resectable or locally advanced pancreatic cancer.

**Variable**	**Hazard Ratio**	***p***
Binary Risk Score		
Low Risk	1.0	--
High Risk	2.71 (1.14–6.48)	0.025
Age		
<60	1.0	--
≥60	0.85 (0.36–2.01)	0.71
Clinical T stage		
1–2	1.0	--
3–4	1.45 (0.52–4.03)	0.48
Clinical N stage		
0	1.0	--
1	0.59 (0.23–1.55)	0.29
Radiation		
Negative	1.0	--
Positive	0.41 (0.16–1.08)	0.070
Surgery		
No	1.0	--
Yes	1.01 (0.39–2.62)	0.98
